# Draft Genome Sequence of the Extremely Halophilic *Bacillus* sp. Strain SB49, Isolated from a Salt Crystallizer Pond of the Little Rann of Kutch, India

**DOI:** 10.1128/genomeA.00869-13

**Published:** 2013-10-17

**Authors:** Kamal Krishna Pal, Rinku Dey, Manesh Thomas, Dharmesh Sherathia, Trupti Dalsania, Ilaxi Patel, Kinjal Savsani, Sucheta Ghorai, Sejal Vanpariya, Bhoomika Sukhadiya, Mona Mandaliya, Rupal Rupapara, Priya Rawal

**Affiliations:** Microbiology Section, Directorate of Groundnut Research, Junagadh, Gujarat, India

## Abstract

Here we report the draft whole-genome sequence (3.72 Mbp) of *Bacillus* sp. strain SB49, an extremely halophilic bacterium isolated from a salt crystallizer pond of the Little Rann of Kutch in India. Unraveling the genome of this organism will facilitate understanding and isolation of the genes involved in imparting extreme osmotolerance.

## GENOME ANNOUNCEMENT

An extremely halophilic and endospore-forming bacterium, *Bacillus* sp. strain SB49 (16S rRNA GenBank accession number JF802167), was isolated from a salt crystallizer pond of the Little Rann of Kutch, Gujarat, India. This organism grows optimally at 15% NaCl (range, 0 to 30%) concentration in medium at a temperature of 37°C and pH 7.5 (range, 5 to 9). The genome of *Bacillus* sp. strain SB49 was sequenced to understand the mechanism(s) of extreme osmotolerance and to isolate the relevant gene(s).

The genome of *Bacillus* sp. SB49 was sequenced by shotgun as well as mate-paired libraries at Macrogen Inc., South Korea, through Sequencher Tech Pvt. Ltd., Ahmedabad, India. In shotgun sequencing, an average read length of 453 bp was generated from 845,614 reads of 382,673,464 bases. However, average read lengths of 466 bp and 465 bp, respectively, were generated in mate-pair libraries from 155,818 reads of 72,629,111 bases and 152,361 reads of 70,866,553 bases, respectively.

The reads were assembled using GS De Novo Assembler v 2.6 (1). The genome assembly of *Bacillus* sp. SB49 (G+C content of 46.97%) has approximately 138-fold coverage and contains 12 scaffolds of 3,726,330 bp with an average length of 310,527 bp. The scaffolds consist of 44 contigs of 3,709,667 bp with an average length of 84,310 bp. *N*_50_ scaffold lengths of 567,128 bp, with the smallest scaffold of 2,486 bp and the largest scaffold of 1,122,558 bp, were obtained. Similarly, *N*_50_ contig lengths of 121,935 bp, with the smallest scaffold contig of 1,919 bp and the largest scaffold contig of 388,308 bp, were obtained. All assembly data were deposited in the DDBJ/EMBL/GenBank nucleotide sequence database.

The draft genome sequence was annotated by use of the RAST server (2), Glimmer 3 (3, 4), GeneMark (5, 6), the KEGG database (7), tRNAScan-SE (8), RNAmmer (9), and Signal P4.1 (10) for identification of subsystems, genes, pathways, RNAs, signal peptides, etc.

Using the various software programs, we predicted 3,907 coding sequences (CDS), with 3,293,355 bp in the CDS. There were 124 RNA-encoding genes (119 tRNA and 5 rRNA genes) and 416 subsystems. Among the CDS, 2,302 are not in a subsystem (948 nonhypothetical and 1,354 hypothetical), whereas 1,605 CDS (1,529 nonhypothetical and 76 hypothetical) are in a subsystem. RAST annotation also revealed associations of 96 genes in stress responses in this organism, including 11 genes involved in osmotic stress, 2 in osmoregulation, 9 in choline and betaine uptake and betaine biosynthesis, 32 in oxidative stress (6 in protection from reactive oxygen species [ROS], 16 in oxidative stress, 2 in glutathione:nonredox reactions, 6 in redox-dependent regulation of nuclear processes, and 2 in glutaredoxins), 3 in cold shock, 15 in heat shock, 9 in detoxification, and 26 in no subcategory. Similarly, 37, 96, and 9 genes associated with serine-glyoxalate, branched-chain amino acids, and glycerol and glycerol-3-phosphate pathways, respectively, have been mapped. Using the Signal P4.1 server, we also predicted 160 signal peptides in this organism. A total of 1,836 CDS were mapped to different biochemical pathways of KEGG (K00003 to K16703, including KEGG orthologs [KOs]).

We are exploring the genome of *Bacillus* sp. SB49 further to isolate the gene(s) involved in imparting extreme osmotolerance and the ability to sustain life in the salt pans of the Rann of Kutch, India.

### Nucleotide sequence accession numbers.

This whole-genome shotgun project has been deposited at DDBJ/EMBL/GenBank under the accession number ATWS00000000. The version described in this paper is version ATWS01000000.
